# Ultrastructural Analysis of the Large Neuronal Perikarya in an Injured Dentate Nucleus Using an Experimental Model of Hyperthermia-Induced Convulsions: The First Qualitative and Quantitative Study

**DOI:** 10.3390/jcm13185501

**Published:** 2024-09-18

**Authors:** Joanna Maria Łotowska, Marta Borowska, Milena Żochowska-Sobaniec, Krzysztof Sendrowski, Maria Elżbieta Sobaniec-Łotowska

**Affiliations:** 1 Department of Medical Pathomorphology, Faculty of Medicine with the Division of Dentistry and Division of Medical Education in English, Medical University of Bialystok, 15-269 Białystok, Poland; joanna.lotowska@umb.edu.pl; 2 Institute of Biomedical Engineering, Faculty of Mechanical Engineering, Bialystok University of Technology, 15-351 Białystok, Poland; 3 Department of Paediatric Neurology, Faculty of Health Sciences, Medical University of Bialystok, 15-274 Białystok, Poland; milena.zochowska-sobaniec@umb.edu.pl (M.Ż.-S.); krsen@wp.pl (K.S.); 4 Department of Developmental Age Medicine and Paediatric Nursing, Faculty of Health Sciences, Medical University of Bialystok, 15-295 Białystok, Poland; 5 Independent Researcher, Sukienna 9/4, 15-881 Białystok, Poland

**Keywords:** febrile seizures in children, model of hyperthermia-induced seizures, rat cerebellar dentate nucleus, large neuronal perikarya, dark cell degeneration, aponecrosis, ultrastructural qualitative, quantitative study

## Abstract

**Background**: Febrile seizures are a common form of convulsions in childhood, with poorly known cellular mechanisms. The objective of this pioneering study was to provide qualitative and quantitative ultrastructural research on the large neuronal perikarya in the cerebellar dentate nucleus (DN), using an experimental model of hyperthermia-induced seizures (HSs), comparable to febrile seizures in children. **Methods**: The study used young male Wistar rats, divided into experimental and control groups. The HSs were evoked by a hyperthermic water bath at 45 °C for 4 min for four consecutive days. Specimens (1 mm^3^) collected from the DN were routinely processed for transmission electron microscopy studies. **Results**: The ultrastructure of the large neurons in the DN affected by hyperthermic stress showed variously pronounced lesions in the perikarya, including total cell disintegration. The most pronounced neuronal lesions exhibited specific morphological signs of aponecrosis, i.e., dark cell degeneration (‘dark neurons’). In close vicinity to the ‘dark neurons’, the aponecrotic bodies were found. The findings of this qualitative ultrastructural study correspond with the results of the morphometric analysis of the neuronal perikarya. **Conclusions**: Our results may constitute interesting comparative material for similar submicroscopic observations on large DN neurons in HS morphogenesis and, in the future, may help to find potential treatment targets to prevent febrile seizures or reduce recurrent seizures in children.

## 1. Introduction

Over the years, neuropathologists have been searching for an animal experimental model that can depict the morphogenesis of fever-induced seizures, i.e., febrile seizures (also called hyperthermia-induced seizures—HSs; hyperthermic convulsions; fever-induced convulsions; fever-associated seizures), and is sufficiently comparable to human febrile seizures. Seizures induced by fever are still the most common form of convulsions in childhood, making them one of the most common problems in pediatric neurology. It is assumed that febrile seizures, especially repeated febrile seizures, constitute the most prevalent kind of epilepsy and neurological illness in infants and young children [1,2,3,4,5,6,7,8].

Febrile seizures frequently affect children aged six months to five years old, with a 2–5% incidence among children under five years of age and a high occurrence between the 12th and 18th month, with the peak incidence in the 24th month of life. It is assumed that 30–40% of pediatric patients with pathological brain activity caused by febrile seizures will have a recurrence during early childhood [1,3,4,7,8,9,10,11].

The correlation between febrile seizures and epilepsy has been well documented in the neurological literature [3,4,6,7,8,12,13,14,15,16]. However, according to some authors, this correlation is highly variable [5]. Based on the clinical features and prognostic evaluation, it has been emphasized that even though simple febrile seizures are usually mild, children with complex febrile seizures are at risk of subsequent epilepsy later in life, including, e.g., drug-resistant epilepsy of the temporal lobe (TLE), associated with mesial temporal sclerosis [5,6,7,13,14,15,16,17]. It has been reported that human infants and immature rodents that have experienced complex febrile seizures have a high risk of subsequent temporal lobe epilepsy [5,7,18].

However, the causes of febrile seizures and the mechanisms underlying the subsequent epileptogenesis remain unknown.

We would like to emphasize here that our current knowledge of the cellular mechanisms underlying HSs gained from animal models evaluated at the level of transmission electron microscopy is very scarce. Therefore, their detection could help design the morphological pattern of febrile seizures in humans and elucidate their potential impact on epilepsy.

As shown in the neuropathological literature, one such CNS structure, constituting a prominent target of such abnormalities in the epileptic brain, is the cerebellar dentate nucleus (DN), in addition to the ammonal cortex of the hippocampal gyrus. Unfortunately, so far, the DN has not been the subject of submicroscopic studies in terms of experimental hyperthermic-induced seizures (HSs) derived from animal models. Since the cerebellum, especially the cerebellar cortex and the cerebellar dentate nucleus, has an inhibitory effect on the motor component of an epileptic seizure, damage to this CNS structure, e.g., in the course of febrile seizures, deprives the body of natural protection in states of seizure readiness [19,20].

It is assumed that the dentate nucleus, the largest and, phylogenetically, the most recent of the cerebellar nuclei located in the cerebellar white matter, plays a part as a major relay center between the cortex and the other part/parts of the brain. It is worth mentioning that the DN receives afferents from the premotor cortex and supplementary motor cortex (via the pontocerebellar system), while its efferents project via the superior cerebellar peduncle through the red nucleus to the ventrolateral thalamus (crossing over at the pontomesencephalic junction). It is responsible for the planning, initiation, and also the control of volitional movements. Morphologically, the population of dentate nucleus neuronal cells can be divided into large and small neurons. The former have large and round somata, long dendrites, and are targets of a number of various extracerebellar systems. Topologically, large neurons can be classified as central and border neurons. The latter, on the other hand, have a small cell body, short dendrites, and belong to the functional class of interneurons [19,21,22,23,24,25,26,27].

Taking the above into account, the aim of the current work was to conduct descriptive, qualitative, ultrastructural research on the large neuronal perikarya in the DN, extended through quantitative analysis using an experimental model of hyperthermia-induced seizures in young rats, developed in our center [28]. This is especially important as, according to some authors, the developing brain is particularly sensitive to hyperthermic stress [18,28,29,30,31]. The present study was inspired by our earlier ultrastructural findings on the morphology of advanced neurodegenerative lesions within the population of pyramidal neurons in the hippocampal CA1 and CA3 sectors in the experimental model of HS previously mentioned [32].

We believe that elucidating of the sequence of morphological events within the brain structures most sensitive to post-seizure changes, using various microscopic techniques, particularly transmission electron microscopy (TEM), could provide a valuable reference in future research into the morphogenesis and progression of febrile seizures in pediatric patients.

We also hope that our results may contribute to identifying potential treatment targets to prevent febrile seizures or reduce recurrent convulsions in children.

## 2. Materials and Methods

### 2.1. Animals

A retrospective electron-microscopic analysis of the large neurons in the rat cerebellar dentate nucleus, both in an experimental model of febrile seizures and in a control group, was conducted at the Department of Medical Pathomorphology, Medical University of Bialystok.

The experiment involved twelve young male Wistar rats aged 22–30 days, which were divided into two groups: experimental (seven rats) and control (five rats). The animals were pre-selected based on standard pharmacological screening tests. All procedures were performed in strict accordance with the Helsinki Convention Guidelines for the care and use of laboratory animals. The study was approved by the Ethical Committee of the Medical University of Bialystok.

### 2.2. Model of Febrile Seizures

The HS group consisted of rats with induced febrile seizures. Hyperthermic stress was evoked by placing the animals in 30 × 30 × 60 cm water bath filled with water maintained 45 °C. The water temperature remained consistent throughout the procedure. The rats were kept in the water for 4 min until convulsions occurred, and were then transferred a separate container lined with lignin. All animals, except for the controls, underwent this procedure for four consecutive days.

Most rats exposed to warm water hyperthermia rapidly exhibited myoclonic jerks, followed by generalized seizures, characterized by vigorous shaking of the head, ears, and upper and lower limbs, along with especially violent tail vibration.

A detailed description of the methodology was presented in our previous paper [28].

### 2.3. Preparation for Transmission Electron Microscopy (TEM)

Seventy-two hours after the last convulsion episode, the rats were anesthetized with Nembutal (25 mg/kg b.m., i.p.). They were then perfused intravitally via the left heart chamber into the superior aorta, with simultaneous clamping of the descending aorta and incision of the right atrium. A fixative solution (approximately 200 mL/animal) containing 2% paraformaldehyde (f. Sigma) and 2.5% glutaraldehyde (f. Serva) in 0.1 M cacodylate buffer (f. Serva), pH 7.4, at 4 °C, was used at a pressure of 80–100 mmHg.

Following perfusion, the brains were removed and the cerebellar hemispheres were dissected and sectioned. Small tissue blocks (1 mm^3^) containing the structure of the dentate nucleus were collected and fixed in the same fixative solution for 24 h. Post-fixation was performed using 1% osmium tetroxide (OsO4) (f. Serva) in 0.1 M cacodylate buffer, pH 7.4, for 1 h. The tissue block were then dehydrated in ethanol and propylene oxide (f. Serva) before being routinely embedded in Epon 812 (f. Serva). Serial sections were obtained using a Reichert ultramicrotome (Reichert Ultracut S) to obtain semithin sections. The semithin sections were stained with 1% methylene blue (f. POCH) in 1% sodium borate (f. POCH) and preliminarily examined under a light microscope to select Epon blocks contained large neurons of the DN. Selected blocks were further sectioned using a Reichert ultramicrotome equipped with a diamond knife to obtain ultrathin sections (70–80 nm), which were placed on 200-mesh grids. Then, ultrathin sections were contrasted with uranyl acetate (f. Serva) and lead citrate (f. Serva) and examined with a transmission electron microscope (Opton EM 900, Zeiss, Oberkochen, Germany) and photographed using a TRS camera (CCD—Camera for TEM 2K inside).

The material from control group’s cerebellar dentate nucleus was processed using the same techniques as for the experimental group (further methodological details can be found in our previous reports [33,34]).

### 2.4. Measurement and Quantitative Analysis of the Large Neuronal Perikarya in the Dentate Nucleus (DN)

Fifty randomly selected images of the perikaryal, observed under 12,000× magnification, were taken from each study group. The regionprops function (https://www.mathworks.com/help/images/ref/regionprops.html, accessed on 11 April 2024) in the Matlab software package was used to measure the following parameters: Area, Circularity, MinFeretDiameter and MinorAxisLength. The Area parameter represents the actual number of pixels in the region of interest (ROI). The Circularity parameter indicate how close the ROI is to a perfect circle, with a value of 1 representing a perfect circle and values less than 1 representing other shapes. The MinFeretDiameter parameter is the minimum Feret diameter, measured as the shortest distance between any two boundary points on opposite vertices of the convex hull surrounding the ROI. The MinorAxisLength parameter represents the length (in pixels) of the auxiliary axis of the ellipse that has the same normalized second central moments as the ROI.

The methodology of the morphometric study was prepared based on Zhao et al. (2010) and Girardet et al. (2010) [35,36].

### 2.5. Statistical Analysis

The data were analyzed using Python-based statistical and visualization libraries, including NumPy (https://numpy.org/, accessed on 11 April 2024), SciPy (https://scipy.org/, accessed on 11 April 2024), and Matplotlib (https://matplotlib.org/, accessed on 11 April 2024). A *t*-test was used for statistical analysis. Values were presented in plots as the mean ± standard deviation (SD), with significance considered at *p* < 0.05.

## 3. Results

The current electron microscopic studies on the HS experimental model revealed numerous interesting neuronal abnormalities, primarily affecting the perikarya and dendrites of the large neuronal cells in the dentate nucleus.

**Figure 1:**
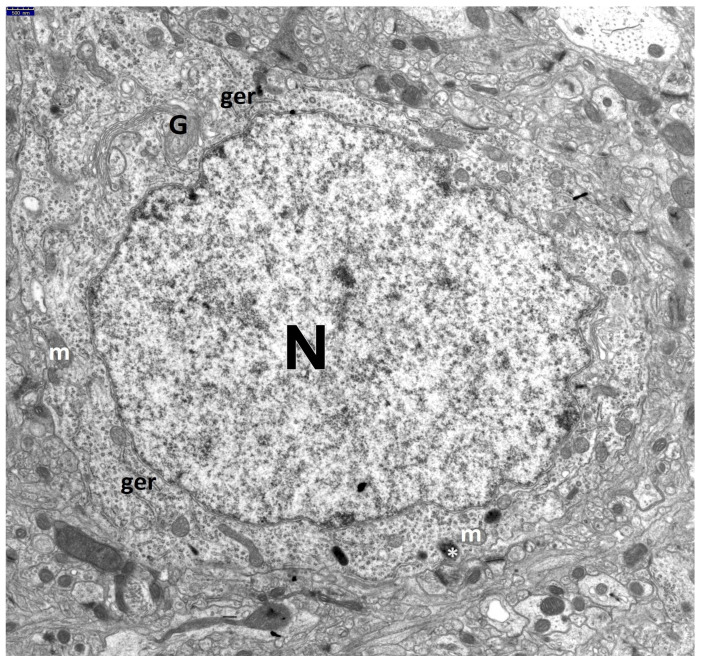
Electronograms demonstrating a well-preserved, round perikaryon of the large neuron in the dentate nucleus in the control group. Cell nucleus (N) is large and bright. In the karyoplasm, evenly dispersed cloud-like clusters of euchromatin are visible. Within the hyaloplasm, well-preserved intracellular structures can be seen: channels of granular endoplasmic reticulum (ger) and quite numerous clusters of polyribosomes; Golgi apparatus (G)—juxtanuclearly located, consisting of parallel smooth channels and cisternae as well as small vesicles; small, oval, mostly elongated mitochondria (m) with a typical two-layered membrane arrangement, the inner one forming fine crests, and a slightly granular matrix. At the periphery of the cell, delicate dense bodies (*) are visible—small bodies 0.1–0.5 µm in diameter, surrounded by a single membrane, which may correspond to primary lysosomes. Scale bar: 500 nm.

The ultrastructural qualitative changes observed in the perikarya of these neurons in all HS animals, compared to the control group (Figure 1), varied in severity, ranging from discrete alterations to complete cell disintegration.

**Figure 2:**
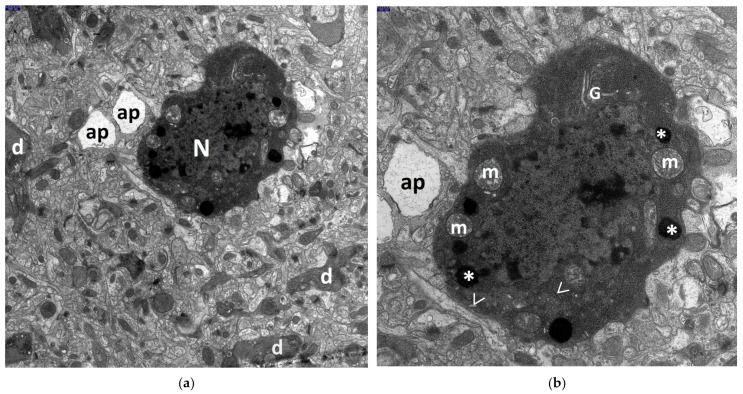
Electronograms demonstrating a morphological view of the perikaryon of the large neuron in the dentate nucleus showing very distinct features of aponecrosis (so called ‘dark neuron’) in the HS group. The aponecrotic cell is shrunken, oval in shape, very dark, nearly black, with cytoplasm demonstrating distinct degeneration and disintegration, with the result that the vast majority of individual endoplasmic structures cannot be distinguished; characteristic dense structures, the so-called dense bodies (*), mostly markedly enlarged (some of them fused together), are clearly visible only within the condensed cytoplasm of the degenerated perikaryon. The cell nucleus (N) showing pronounced degeneration, with features of substantial homogenization, merges with the cytoplasmic contents; the nucleolus shows signs of disintegration. The cytoplasm is filled with a dark homogeneous microgranular material probably derived from disintegrated polysomes. In places, within dark cytoplasm, contours of enlarged mitochondria (m), with features of condensed configuration, disintegrating elements of the Golgi apparatus (G), and shadows of disintegrating ger channels (>) can be recognized. The surrounded neuropil elements are markedly swollen in places, especially the astrocytic processes (ap), whereas the dendritic processes (d) are mostly dark and shrunken. Scale bar: 500 nm (**a**) and 250 nm (**b**).

**Figure 3:**
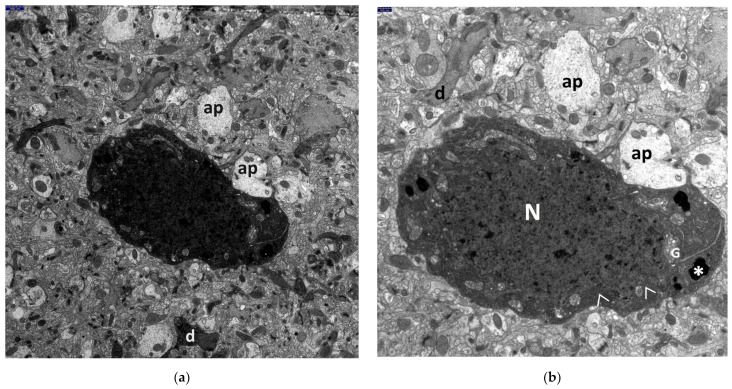
The ultrastructure of another perikaryon of the large neuron in the dentate nucleus closely resembles the one described in Figure 2a,b and demonstrates pronounced features of dark cell degeneration in the HS group. The aponecrotic neuron is shrunken, oval in shape, with the cytoplasm containing disintegrating endoplasmic structures, e.g., elements of Golgi apparatus (G), mitochondria, ger channels (>); markedly enlarged dense bodies (*) can be seen on the cell periphery. In places, injured neuropil elements—markedly swollen astrocytic processes (ap) and shrunken, mostly dark dendritic processes (d) surround the cell nucleus (N). Scale bar: 1000 nm (**a**) and 500 nm (**b**).

**Figure 4:**
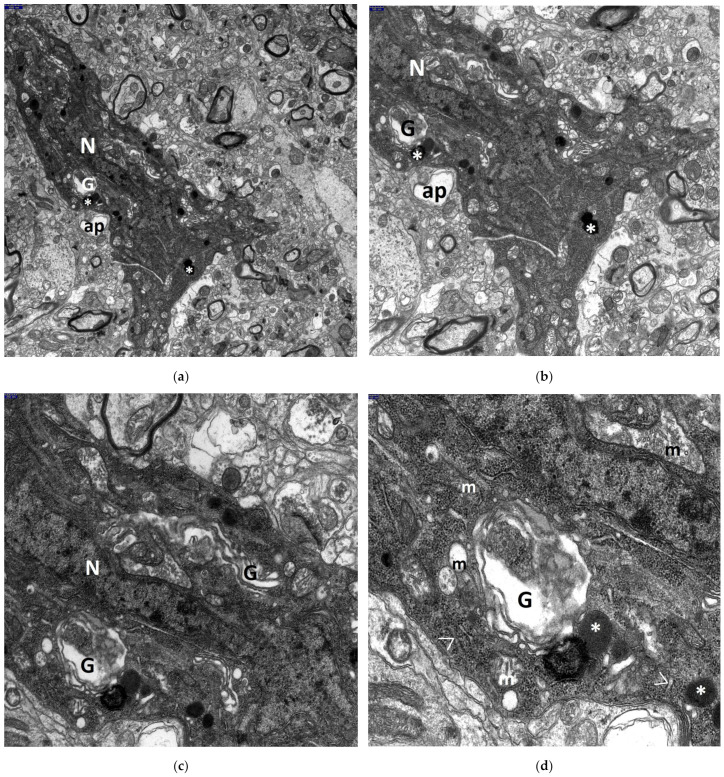
Electronograms showing morphological details within a large fragment of degenerated neuronal perikaryon in dentate nucleus, illustrating the immediate stage preceding the formation of the dark aponecrotic neuron, in the HS group. The neuronal perikaryon is distinctly shrunken, triangular in shape, with considerably increased electron density of the cytoplasm, containing abundant agglomerations of polysomes with accompanying short fragments of ger channels (>). Distinctly dilated channels and cisterns of the Golgi apparatus (G) and pleomorphic mitochondria (m)—some are swollen, some dark with features of condensed configuration are visible; some dense bodies (*) are markedly enlarged. In places, neuropil elements, mainly astrocytic processes (ap), adhering to the perikaryon show features of swelling. Cell nucleus (N) with a ‘homogenized’ appearance. Scale bar: 1000 nm (**a**), 500 nm (**b**), 250 nm (**c**), and 100 nm (**d**).

**Figure 5:**
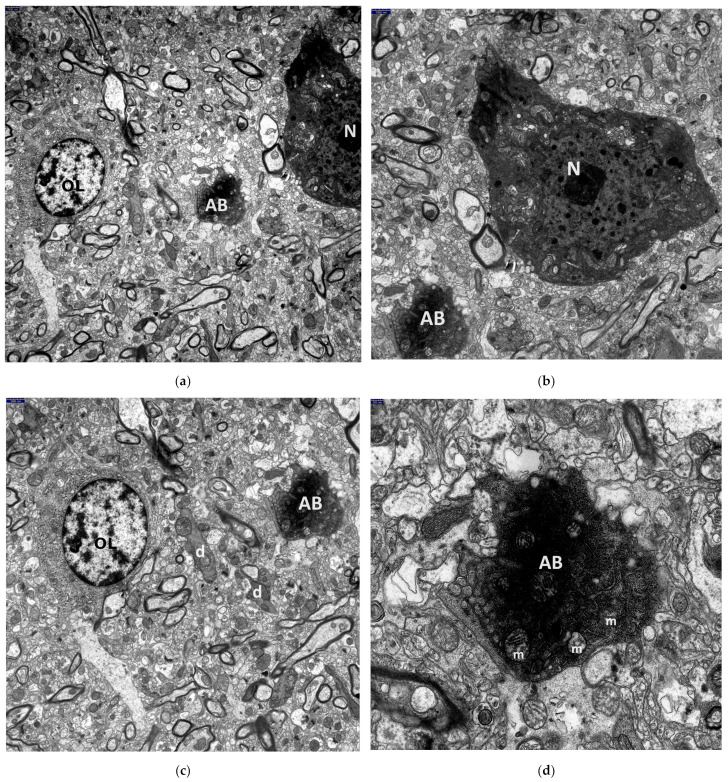
Electronograms demonstrating interesting details of aponecrotic body morphogenesis in dentate nucleus in the HS group. A very dark, almost black aponecrotic body (AB) is separated from a nearby aponecrotically changed neuronal perikaryon; its degenerated nucleus (N) contains substantially enlarged, centrally located nucleolus. Numerous enlarged, disintegrated dark mitochondria (m) are visible in the condensed cytoplasm of the AB. The AB is surrounded by markedly swollen neuropil elements. Moreover, electronograms (**a**,**c**) clearly demonstrate the image of a well-preserved oligodendrocyte (OL). In places, clearly visible are sections through dark, shrunken dendritic processes (**d**). Scale bar: 1000 nm (**a**), 1000 nm (**b**), 1000 nm (**c**), and 250 nm (**d**).

**Figure 6:**
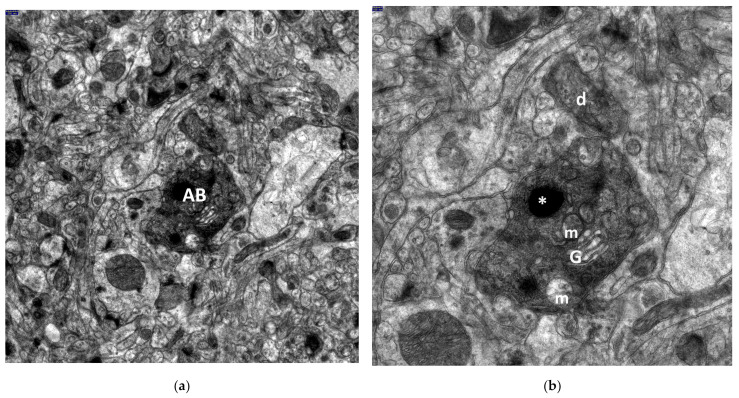
Electronograms clearly showing a very dark aponecrotic body (AB) of dentate nucleus in the HS group. In close vicinity, a section through dark dendritic process (d) is present. Two dense bodies, one of markedly increased size (*), possibly corresponding to primary lysosomes are present within the AB; nearby an accumulation of degenerated mitochondria (m), mainly with a condensed configuration. In the lower part of the aponecrotic body, distinctly dilated channels of Golgi apparatus (G) and a single, enlarged and markedly swollen mitochondrion are also well visible. Scale bar: 250 nm (**a**) and 100 nm (**b**).

**Figure 7:**
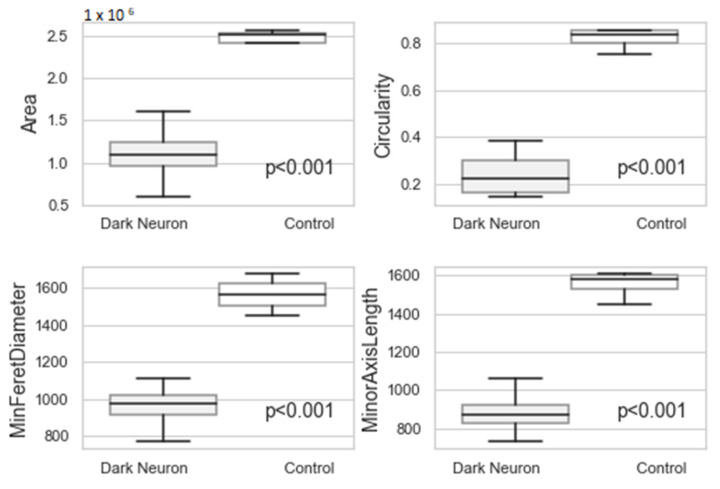
The value of parameters Area, Circularity, MinFeretDiameter, and MinorAxisLength. Data are compared between Control and Dark Neuron in HS groups. Data in box plots are represented by lower quartile, mean, and upper quartile, whereas whiskers represent minimum and maximum values. The significance level was established as *p* < 0.05.

**Figure 8:**
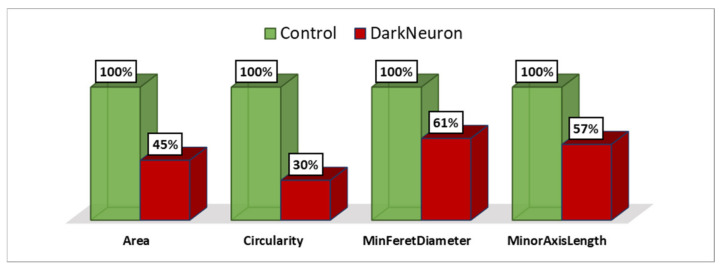
The %parameters (in pixels) for Area, Circularity, MinFeretDiameter, and MinorAxisLength between Control and Dark Neuron in HS groups.

The most advanced neuronal lesions in rats exposed to hyperthermic stress exhibited distinct morphological signs of aponecrosis. The submicroscopic image of aponecrotic neurons displayed clear features of dark cell degeneration, characterized by dark or very dark, often nearly black ischemic cells—referred as ‘dark neurons’, as documented in a series of electronograms (Figure 2, Figure 3, Figure 4, Figure 5 and Figure 6). However, the results of the morphometric (i.e., ultrastructural quantitative) analysis of large neuronal perikarya of the dentate nucleus are summarized in Table 1 and in Figure 7 and Figure 8.

The perikarya of aponecrotic neurons were typically shrunken and varied in shape, most often appearing oval, less frequently triangular, with a significantly increased density of the cytoplasm and karyoplasm. The cytoplasm frequently exhibited features of several degeneration, including disintegration (Figure 2a,b and Figure 3a,b). It is noteworthy to mention that several dark, dense structures were present within both the karyoplasm and the cytoplasm of the nervous cell.

The morphogenesis of dark cell neuronal degeneration is well documented in a series of electronograms (Figure 4a–d), which depict ultrastructural details illustrating the stage just before the formation of the characteristic dark neuron. The perikaryon of the large neuron, which is distinctly shrunken, contains a completely degenerated, “homogenized” nucleus and cytoplasm with significantly increased electron density. The cytoplasm is filled with dark, homogenous, microgranular material, likely derived from disintegrated polysomes and from broken channels of the granular endoplasmic reticulum (GER) (Figure 4d). Additionally, the electronograms clearly show morphological changes, such as pronounced dilatation of Golgi apparatus channels and cisterns, forming large vacuolar spaces (Figure 4c,d), as well as varying degrees of mitochondrial destruction and the presence of significantly enlarged, dense bodies (Figure 4a–d).

An interesting phenomenon observed within the large degenerated neuronal perikarya is worth noting. The vicinity of markedly swollen mitochondria, characterized by an increased electron-translucent matrix and residual cristae at their periphery (often with sometimes damaged outer mitochondrial membrane), also included shrunken mitochondria displaying a distinctly condensed configuration. This was accompanied by the presence of enlarged dense bodies (Figure 3a,b and Figure 4a–d).

In close proximity to aponecrotically damaged neuronal perikarya, fragmented cell bodies known as aponecrotic bodies were observed. The characteristic appearance of these aponecrotic bodies is shown in Figure 5a–d and Figure 6a,b.

The presence of such advanced aponecrotic changes in the large neurons of the dentate nucleus often led to their complete disintegration and irreversible dentate neuronal loss.

Frequently, both dark, degenerated neuronal perikarya and aponecrotic bodies were surrounded by markedly damaged neuropil elements of the dentate nucleus, primarily swollen astrocytic processes (Figure 2a,b, Figure 3a,b, Figure 4a,b, and Figure 5d).

Nearby dendritic processes were usually shrunken, dark, or very dark and exhibited significant degeneration of dendroplasm (Figure 2a, Figure 3a, Figure 5c, and Figure 6b). Less frequently, clearly swollen dendritic processes were observed. A detailed morphological analysis of neuronal dendrites affected by hyperthermic stress in the dentate nucleus will be presented in the subsequent study.

It should be noted that in close proximity of dark shrunken neurons, some neuronal and glial cells exhibited relatively well-preserved morphology (Figure 5a,c).

The neurodegenerative changes in the DN were accompanied by significant damage to the blood–brain barrier’s structural components, similarly to the findings previously reported in the hippocampal gyrus cortex in an analogous experimental model of febrile seizures [37,38,39].

The morphometric analysis revealed that the Area, Circularity, MinFeretDiameter and MinorAxisLength of large neuronal perikarya were higher in the control group compared to the dark neuron in the HS group (Table 1 and Figure 7 and Figure 8). The correlation coefficients between the analyzed parameters were above 0.92 (Table 2).

The Circularity parameter proved to be the most distinctive. Large neurons in the control group were more circular than dark neurons in the HS group. Additionally, the surface area of the dark neurons in the HS group was smaller compared to the unchanged large neurons. The diameter of the aponeurotic degenerated perikarya in the HS group was significantly smaller than that of the large neurons in the control group.

## 4. Discussion

While there are many clinical and epidemiological studies on febrile seizures in children, there are no comparable neuropathological reports available to serve as a reference for our current research. Only a few morphological studies using electron microscopy have been conducted, including those from our center, focusing on the microscopic examination of selected CNS structures in an experimental model of febrile seizures in young rats.

The brain maturity of male Wistar rats aged 22–30 days, used in our experimental model of febrile seizures, may correspond to that of 1- or 2-year-old children [28,29]. More recent reports suggest it could be equivalent to 3–5 years old children [31,40]. However, the exact age equivalence between rat and human brain remains uncertain [29].

This study presents the first attempt to explore cellular mechanisms through both qualitative and quantitative ultrastructural assessment of large neuronal perikarya in the dentate cerebellar nucleus of rats damaged due to hyperthermic convulsions.

We found that heat stress-induced HS produced distinct and easily recognizable morphological alterations in the large neurons of rat DN. These changes included lethal dark cell degeneration, resulting in neuronal loss within the explored cerebellar structure. The most severe neuronal injuries observed in animals exposed to febrile seizures displayed clear ultrastructural features of aponecrosis. This was characterized by the numerous distinctly shrunken, dark or very dark degenerated ischemic cells, referred to as ‘dark neurons’, and the presence of several dark, dense structures within the karyoplasm and cytoplasm of the nerve cells. The cell nuclei of lethally damaged neuronal perikarya were filled with a ‘homogenized’ karyoplasm, while the cytoplasm was condensed and substantially degenerated, leading to neuronal death and complete disintegration. An interesting phenomenon was the appearance of characteristic fragments of cell bodies, known as ‘aponecrotic bodies’, in close proximity to dark neurons. It is noteworthy that 40 years ago, in neuropathology, the ultrastructural appearance of nerve cells exhibiting aponeurosis due to neuronal death in ‘epileptic’ brain damage was referred to as neuronal soma necrosis or ‘dark cell degeneration’ [41].

We observed a close correlation between the qualitative morphological changes in the perikarya of ‘dark shrunken neurons’ in the rat DN and their quantitative morphometric assessment. The morphometric analysis indicated that the circularity parameter was the most characteristic of these perikarya.

The descriptive ultrastructural changes observed in the large neurons of the dentate nucleus in HS-exposed animals clearly demonstrate that this region of the brain is significantly involved in the cellular mechanisms of neuronal damage caused by experimental hyperthermic convulsions. These findings are consistent with those reported by other studies on the neurons in the cerebellum and brain-stem of rats exposed to heat [30,42], as well as in hippocampal neurons in rats subjected to a model of febrile convulsions [31,43].

It is worth noting that the morphological abnormalities observed in the current study closely resemble changes seen in neuronal cell perikarya in other brain regions analyzed in our earlier studies using TEM. These include the hippocampal CA1 and CA3 sectors [32] and in the neocortex of the temporal lobe in a similar rat experimental model of febrile seizures [38].

The submicroscopic abnormalities observed in large dentate neurons, including their perikarya and surrounding neuropil elements, in the experimental hyperthermia model in young rats—believed to be comparable to febrile convulsions in children—indicate a profound disturbance in intracellular processes. These transformations lead to dark cell degeneration, lethal damage, and the formation of numerous characteristic ‘dark’ ischemic cells, referred as ‘dark neurons’. Specifically, the degranulation and disintegration of GER channels and polysomes resulted in abnormalities in protein production, while mitochondrial destruction inhibited oxidative phosphorylation. Damage to the Golgi apparatus disrupted detoxification and secretory processes, and disturbances in maintaining the morphological integrity of the cell, exemplified by the detachment of ‘aponecrotic bodies‘ from the perikaryon, led to nerve cell disintegration.

The pathomechanism underlying structural damages to the cerebellar dentate nucleus in the febrile convulsion model is undoubtedly complex and requires further ultrastructural descriptive and morphometric studies by other research centers investigating similar topics.

We believe that in addition to the direct effects of elevated temperature on the large neurons of the dentate nucleus and surrounding neuropil elements, the secondary pathogenic effects of CNS ischemia and tissue edema—resulting from similar damage to the structural components of the blood–brain barrier—should also be considered. This indirect vascular factor is supported by our previous ultrastructural findings on the morphological elements of the blood–brain barrier in other CNS regions, such as the hippocampal CA1 and CA3 sectors [37,39] and temporal lobe neocortex in a similar febrile convulsion model [38,44]. We speculate that the vascular factor may exacerbate the morphological abnormalities initiated by hyperthermia in the cellular elements of the cerebellar dentate nucleus, although this hypothesis require further in-depth submicroscopic studies.

We hope that the current neuropathological study of large neurons in the cerebellar dentate nucleus, using TEM in the experimental HS, will serve as a valuable reference for comparing human febrile seizures with this animal model. Enhanced understanding of the morphogenesis of neuronal abnormalities resulting from experimental febrile seizures, where heat stress-induced hyperthermia causes specific ultrastructural changes in various brain regions, should aid in developing therapies aimed to minimizing brain damage.

## 5. Conclusions

This descriptive ultrastructural study, complemented by a morphometric analysis of the perikarya of large DN neurons in young rats using TEM, demonstrated that HS-induced heat stress produced specific and easily recognizable morphological features of aponecrosis. These features include the presence of characteristic dark neurons and aponecrotic bodies in close proximity. We hope that a better understanding of the cellular mechanisms underlying hyperthermic seizures in this animal model will aid identifying potential treatment targets to prevent or reduce recurrent febrile seizures in children.

## Figures and Tables

**Table 1:** The values (mean ± SD) and %parameters of Area (pixels) (mean and standard deviation), Circularity (mean and standard deviation), MinFeretDiameter (pixels) (mean and standard deviation), and MinorAxisLength (pixels) (mean and standard deviation) of the Control and Dark Neuron in HS groups. The significance level was established as *p* < 0.05.

	Area (%)	Circularity (%)	MinFeretDiameter (%)	MinorAxisLength (%)
Control	2,441,706 ± 184,695 (100)	0.818 ± 0.046 (100)	1563 ± 99 (100)	1553 ± 71 (100)
Dark Neuron in HS group	1,099,657 ± 414,031 (45)	0.245 ± 0.110 (44)	961 ± 141 (30)	885 ± 135 (30)
*p*	<0.001	<0.001	<0.001	<0.001

**Table 2:** Correlation coefficient between analyzed parameters.

	Area	Circularity	MinFeretDiameter	MinorAxisLength
Area	1.00	0.92	0.99	0.99
Circularity	0.92	1.00	0.92	0.95
MinFeretDiameter	0.99	0.92	1.00	0.99
MinorAxisLength	0.99	0.95	0.99	1.00

## Data Availability

The original contributions presented in the study are included in the article, further inquiries can be directed to the corresponding authors.
